# Safety and efficacy of delivering systemic treatments concomitantly with whole-brain radiotherapy: a prospective, observational, single-institution study

**DOI:** 10.37349/etat.2026.1002395

**Published:** 2026-08-07

**Authors:** Maria Protopapa, Kalliopi Platoni, Michalis Psarras, Styliani-Theonymfi Nikoloudi, Zoi Liakouli, Despoina Alexiou, Christina Armpilia, Christos Antypas, Christos Papadimitriou, Vasileios Kouloulias, Anna Zygogianni

**Affiliations:** IRCCS Istituto Romagnolo per lo Studio dei Tumori (IRST) “Dino Amadori”, Italy; ^1^Radiation Oncology and Stereotactic Radiosurgery Center, Mediterraneo Hospital, 116 75 Glyfada, Greece; ^2^Radiation Oncology Unit, Aretaieion Hospital, Medical School, National and Kapodistrian University of Athens, 115 28 Athens, Greece; ^3^Department of Applied Medical Physics, Attikon University Hospital, Medical School, National and Kapodistrian University of Athens, 124 62 Athens, Greece; ^4^Radiation Oncology Department and Clinic, Medical School, University of Ioannina, 445 00 Ioannina, Greece; ^5^Oncology Unit, Aretaieion University Hospital, Medical School, National and Kapodistrian University of Athens, 115 28 Athens, Greece; ^6^Department of Clinical Radiation Oncology, Attikon University Hospital, Medical School, National and Kapodistrian University of Athens, 124 62 Athens, Greece

**Keywords:** whole-brain radiotherapy (WBRT), brain metastases, systemic therapy, concurrent treatment

## Abstract

**Aim:**

The aim of our study was to evaluate the safety and efficacy of delivering systemic treatments concurrently with whole-brain radiotherapy (WBRT).

**Methods:**

A single-institution, prospective observational study was conducted in Athens, Greece, including 99 patients treated with WBRT for brain metastases from September 2017 until October 2019, and with a follow-up period extending to March 2025. The study endpoints included overall survival (OS) for all causes of death, time to intracranial progression (TTICP), and serious acute toxicities.

**Results:**

The median OS from all causes of death was 6 months [95% confidence interval (CI): 4.7–7.3]. Concomitant systemic therapy reduced the risk of death (*p* = 0.005), and the presence of systemic metastases (*p* = 0.009) increased the risk of death for patients with brain metastases treated with whole brain radiotherapy. The median TTICP was 10 months (95% CI: 3.3–16.6), with a more protracted fractionation and a larger number of brain metastases being prognostic of an increase in the TTICP. Acute severe toxicity was observed in 16.2% of patients, with no statistically significant difference between concurrent and no systemic therapy groups, and with no treatment interruptions in patients treated concurrently.

**Conclusions:**

This study showed that there is no serious toxicity from the combination of systemic therapy with WBRT, and that the OS and the TTICP are not compromised with their concurrent delivery.

## Introduction

Brain metastases (BM) have a direct impact on patients’ prognosis and their course of treatment, and affect up to 20% of oncological patients [1]. Historically, whole-brain radiotherapy (WBRT) was the cornerstone of management for all patients with BM [2]. Stereotactic radiotherapy (SRS) is the standard of care for BM patients with limited intracranial disease [3], on the basis of randomized controlled trials (RCTs) showing a statistically significant benefit with SRS over WBRT in terms of neurocognition, without jeopardizing overall survival (OS), at the expense of a lower intracranial control [4–7]. WBRT is still the main treatment for BM patients with a significant intracranial tumor burden. Yet, as SRS requires specific radiotherapy equipment, unavailable in many areas of the world, WBRT remains the only available treatment option for the majority of BM patients in underserved areas [8].

In the last decade, systemic agents have revolutionized the prognosis and quality of life of metastatic patients. These include mainly drugs that target somatic driver mutations and immune modulatory agents. At the time of the initiation of this study, all new systemic agents had been approved based on clinical trials that excluded either all BM patients or accepted only patients with asymptomatic and stable BM after local therapies for their intracranial disease [9–12]. Yet, in clinical practice, these systemic treatments were commonly prescribed also for BM patients, and, at the discretion of the treating physician, they were not stopped during WBRT, or they were initiated concurrently with WBRT, despite possible safety issues. The aim of the study was to evaluate the safety and effectiveness of the administration of systemic agents concurrently with WBRT.

## Materials and methods

An observational prospective study on the safety and efficacy of concomitant administration of systemic treatments with WBRT was conducted at the Aretaieion University Hospital in Athens, Greece, from 2017 to 2025. All patients referred for WBRT to the Radiation Oncology Department of Aretaieion University Hospital from 01 September 2017 until 31 October 2019, who gave their consent to participate in the observational study, were candidates for the study. Indications for WBRT at the time in Greece included patients with BM, both intact and resected, regardless of their number or volume. A limited number of BM were not commonly treated with SRS during the period of the study, as SRS was not available in any public hospital.

Inclusion criteria were adult patients, i.e., ≥ 18 years old, with a biopsy-proven solid tumor diagnosis, a CT or MRI brain confirming BM, and accurate data on the timing of systemic treatment from WBRT. Patients who had surgical excision of BM prior to WBRT were included in the study. Yet, patients with previous radiotherapy treatments or previous neurosurgical operations for other reasons were excluded, to avoid adding up toxicities from multiple brain treatments. Confirmed leptomeningeal disease was an exclusion criterion of the study as it alters patients’ prognosis. The study received approval from the Ethics Committee of Aretaieion University Hospital of Athens, Greece.

WBRT was delivered by a 6 MV linear accelerator with opposed lateral fields to a dose of either 30 Gy in 10 fractions or 20 Gy in 5 fractions. Systemic treatments were given or not concurrently with WBRT at the discretion of the medical oncologist. Demographic data and treatment parameters were collected for all patients. Patient data comprised the primary diagnosis, age at BM diagnosis, biological sex, number of BM, presence of other metastatic sites, and presence of neurological symptoms at first visit at the Radiation Oncology Department. Treatment data comprised WBRT initiation and completion, fractionation, and systemic therapy delivered concomitantly or not with WBRT. Systemic therapy was considered concomitant when given within two weeks of WBRT. Two weeks was chosen as this is considered a common time interval to distinguish between early and late WBRT [13]. Also, two weeks is clearly a time period during which treatment effects can potentially accumulate, so that many RCTs mandate 4–8 weeks from radiotherapy to systemic treatments [14–16]. Patients and treatment data by treatment group, i.e., with or without concomitant systemic therapy, are summarized in Table 1.

**Table 1:** Demographics of the patient population included in the analysis.

Stratification factors		Patients	*N*%
Therapy group	Non systemic	63	63.6%
	Systemic	36	36.4%
Biological sex	Female	42	42.4%
	Male	57	57.6%
PS ≥ 2	No	67	67.7%
	Yes	32	32.3%
Number of brain metastases	No brain metastasis (GTR)	4	4.0%
	1 brain metastasis	26	26.3%
	2–4 brain metastasis	40	40.4%
	> 4 brain metastasis	29	29.3%
Systemic metastases in addition to brain metastases	No	41	41.4%
	Yes	58	58.6%
Neurological symptoms	No	34	34.3%
	Yes	65	65.7%
Lung adenocarcinoma	No	65	65.7%
	Yes	34	34.3%

PS: performance status; GTR: gross tumor resection.

Follow-up was performed by the patient or the immediate relative or other adult who was the patient’s caregiver, accompanying the patient during WBRT, or by visits to the Radiotherapy Unit. Follow-up visits were scheduled to take place every 3 months after WBRT completion to document any event relevant to the treatment of BM. Follow-up imaging with CT or MRI brain, as requested by their medical oncologist, was also recorded whenever available. Intracranial response was reported at each follow-up visit for which imaging could be provided based on the Response Assessment in Neuro-Oncology for BM (RANO-BM) criteria [17]. Data collected comprised date of death, date of intracranial progression, and presence of serious acute toxicity, i.e., grade 3 or higher toxicity, within three months from WBRT. Patients were also monitored for neurological toxicity after 3 months. Follow-up continued until death or until 31 March 2025. Primary end points of the study were OS and serious acute adverse events for the whole patient population and by treatment group. The secondary endpoint of the study was time to intracranial progression (TTICP).

Statistical analyses were conducted using SPSS version 27.0 (SPSS Inc., Chicago, IL, USA). Continuous variables were expressed as mean ± standard deviation and categorical variables were expressed as percentages. All *p*-values < 0.05 were considered statistically significant. A chi-square (*χ*^2^) test was used to determine whether two categorical variables are related to each other. Time-to-event outcomes were estimated using Kaplan-Meier curves. The log-rank test was used to assess the effect of the predictors on OS and TTICP. OS is defined as the time from the time of WBRT to death for any reason or lost to follow-up. TTICP is defined as the time from WBRT to imaging confirmation of intracranial progression. Intracranial progression included progression of previously diagnosed lesions and diagnosis of new lesions. Cox Regression analysis was performed to determine factors predicting OS and TTICP. Continuous data are reported as median, and categorical data are reported as number.

## Results

From September 2017 to October 2019, among patients referred to the Radiation Oncology Unit of Aretaieon University Hospital for WBRT, 102 patients fulfilling the inclusion criteria of the study gave their informed consent. Three out of 102 patients consenting to participate in the study did not receive WBRT because of hospitalization during the waiting period to initiate WBRT. These patients were excluded from the analysis as they could not contribute to either the safety or efficacy of the combined treatment over WBRT alone. Two patients did not complete all fractions of WBRT because of the need for hospitalization. These patients were included in the study to monitor toxicity from the treatment. Of the 99 patients included in the analysis, 57 were males and 42 were females, consisting of 57.6% and 42.4% of the patient population, respectively. Systemic disease was present in 58 (58.6%) patients. The median age of the patient population was 64 years, ranging from 37 to 87 years. 32 (32.3%) patients had a PS ≥ 2 and 63 (63.6%) patients were treated for symptomatic BM.

WBRT was administered in five fractions in 20 (20.2%) patients and as a ten-fraction regimen in 79 (79.8%) patients. Eight (8.1%) patients had previously undergone gross or subtotal resection for a BM. A total of 36 (36.4%) patients received systemic therapy concurrently with WBRT, with some patients receiving drugs from more than one class of antineoplastic agents. An immune checkpoint inhibitor (ICI), a tyrosine kinase inhibitor (TKI), hormone treatment, anti-HER2 drugs, and chemotherapy were prescribed in eight, three, eleven, five and 16 patients, respectively. Table 1 shows the main demographic characteristics of our study population. During a median follow-up period of 6 months, with a range of 1 to 85 months, eighty-three patients (83.8%) died, irrespective of the cause of death; 14 (14.1%) patients were lost to follow-up, and two patients (2.02%) were alive. The median OS time was 6 months [95% confidence interval (CI): 4.7–7.3].

**Figure 1:**
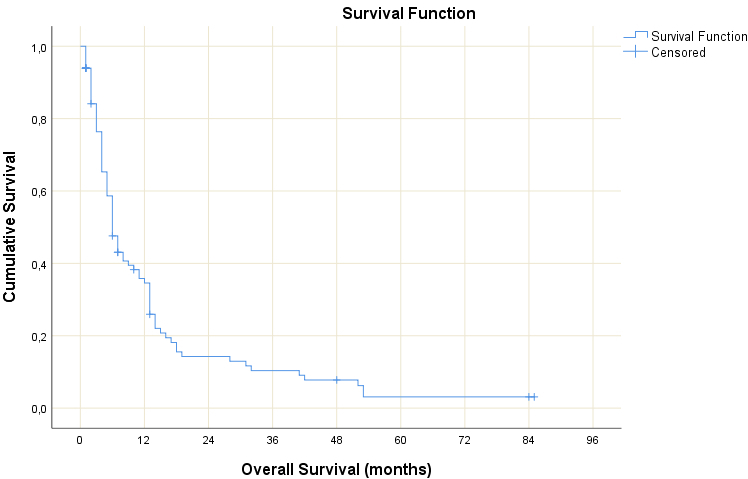
**Kaplan-Meier plot of the overall survival of the study population for all causes of death.** Censored cases are indicated by “+” symbols. The y-axis represents cumulative survival probability, and the x-axis shows overall survival in months.

**Figure 2:**
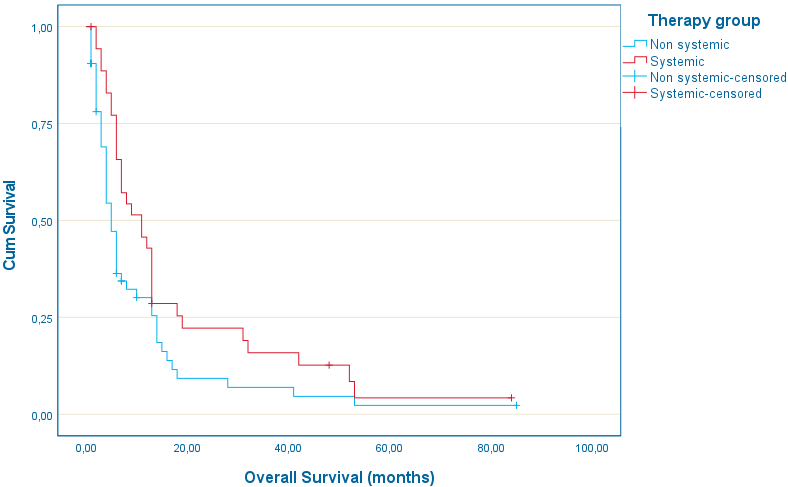
**Kaplan-Meier curves for overall survival by therapy group.** Patients receiving systemic therapy (red line) and non-systemic therapy (blue line) show differences in overall survival over time. Censored cases are indicated with “+” symbols. The vertical axis represents cumulative survival probability, and the horizontal axis shows overall survival in months.

Figure 1 depicts the Kaplan-Meier plot for the OS in months of the study population for all causes of death. Figure 2 depicts the Kaplan-Meier curves for the OS by therapy group. The mean survival time for patients receiving systemic therapy was 17.9 months (95% CI: 11.1–24.8), while for those receiving non-systemic therapy it was 10.6 months (95% CI: 6.3–15.0). The median survival time was 11.0 months (95% CI: 5.2–16.8) for the concurrent systemic therapy group and 5.0 months (95% CI: 3.8–6.2) for the non-concurrent systemic therapy group. The log-rank (Mantel-Cox) test indicated a statistically significant difference in OS between the two therapy groups [*χ*^2^ (1) = 4.693, *p* = 0.030], suggesting that patients receiving systemic therapy concurrently had significantly longer survival compared to those in the non-concurrent therapy group.

The independent variables chosen for the Cox proportional hazard model for all causes of death were a) concomitant systemic treatment, b) biological sex, c) PS ≥ 2, d) age, e) number of BM (0, 1, 2–4, > 4), f) presence of systemic metastases, g) presence of neurological symptoms, and h) lung adenocarcinoma primary. Table 2 lists the model parameter estimates. Of the above independent variables, only concomitant systemic treatment and the presence of systemic metastases had a statistically significant correlation with risk of death from all causes. In the concomitant systemic treatment group, the risk of death from all causes was reduced by 52.5% compared to the non-systemic therapy group (*p* = 0.005). The presence of systemic disease at WBRT initiation was associated with an increased death rate from all causes of 99.9% compared to its absence (*p* = 0.009).

**Table 2:** Model parameter estimates of the Cox proportional hazard model for all causes of death.

Stratification factors	B	SE	Wald	df	*p*-value	HR	95% CI for HR	
							**Lower**	**Upper**
Therapy group	–0.744	0.266	7.839	1	0.005	0.475	0.282	0.800
Biological sex	0.167	0.261	0.410	1	0.522	1.182	0.709	1.972
PS ≥ 2	0.331	0.242	1.869	1	0.172	1.393	0.866	2.239
Number of brain metastases	0.022	0.128	0.030	1	0.863	1.022	0.796	1.313
Systemic metastases in addition to brain metastases	0.693	0.264	6.880	1	0.009	1.999	1.191	3.355
Neurological symptoms	0.113	0.242	0.218	1	0.641	1.120	0.696	1.801
Lung adenocarcinoma	–0.361	0.262	1.899	1	0.168	0.697	0.417	1.165

B: regression coefficient (beta); SE: standard error; df: degrees of freedom; HR: hazard ratio; CI: confidence interval.

**Figure 3:**
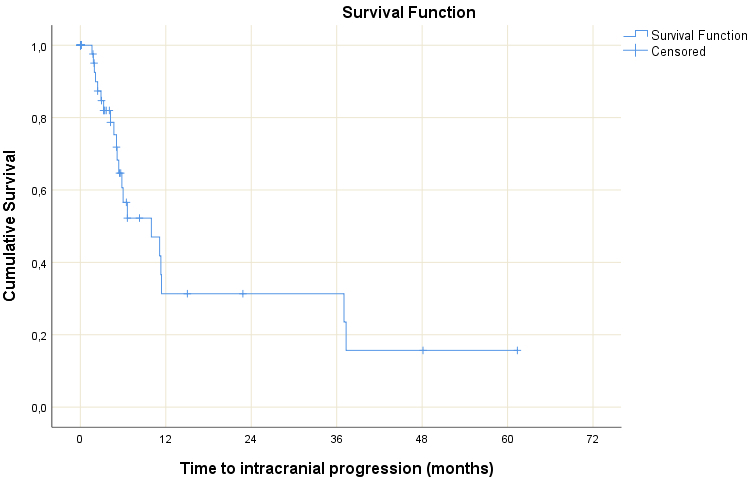
**Kaplan-Meier plot for the time to intracranial progression of the study population.** Censored cases are indicated by “+” symbols. The y-axis represents cumulative survival probability, and the x-axis shows time to intracranial progression in months.

**Figure 4:**
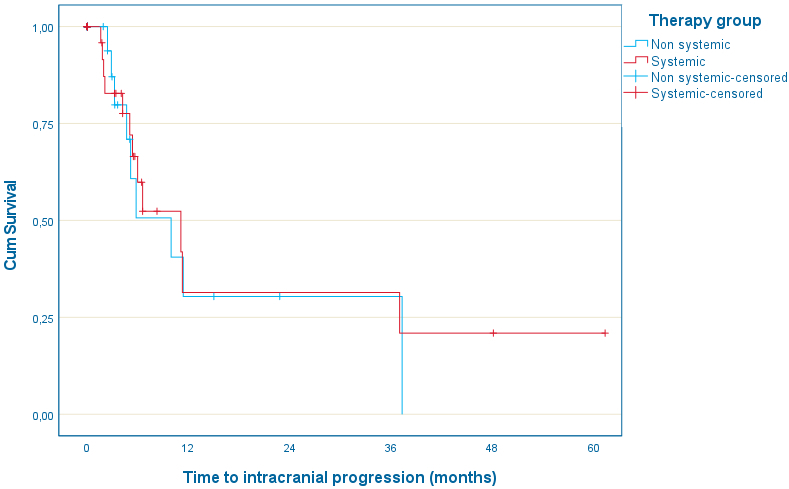
**Kaplan-Meier curves for time to intracranial progression by therapy group.** The plot compares cumulative survival without intracranial progression between patients receiving systemic therapy (red line) and non-systemic therapy (blue line). Censored cases are indicated by “+” symbols. The y-axis represents cumulative survival probability, and the x-axis shows time to intracranial progression in months.

Twenty-one patients (21.2%) had intracranial progression confirmed by imaging exams (MRI or CT), whereas 78 (78.8%) were censored cases. Imaging was not available for most patients, as many died before 3 months follow-up, and others could not provide a brain imaging follow-up exam for a variety of reasons, such as reluctance of treating physicians to ask for more exams in poor-performance patients, or hospitalization for systemic disease progression. The median TTICP was 10 months (95% CI: 3.3–16.6). Figure 3 depicts the Kaplan-Meier plot for the TTICP of the study population in months. Figure 4 depicts the Kaplan-Meier plot for the TTICP in months by therapy group. The median survival time was 11.1 months (95% CI: 3.6–18.7) for the systemic therapy group and 10.0 months (95% CI: 3.0–17.0) for the non-systemic therapy group. The log-rank (Mantel-Cox) test indicated no statistically significant difference in the TTICP between the two groups [*χ*^2^ (1) = 0.057, *p* = 0.811].

As independent variables for the Cox proportional hazard model for the TTICP, we chose a) therapy group (non-systemic, systemic), b) number of BM (0, 1, 2–4, > 4), c) co-existence of systemic disease (no, yes), and d) fractionation (5 × 400 cGy, 10 × 300 cGy). Table 3 lists the model parameter estimates. Of the independent variables under study, the number of BM and the fractionation significantly affected the TTICP. The 10 × 300 cGy group had an increased TTICP of 391.7% compared to the 5 fractions group (*p* = 0.024). A larger number of BM is found to be associated with a longer TTICP (*p* = 0.036).

**Table 3:** Model parameter estimates for the Cox proportional hazard model for time to intracranial progression.

Stratification factors	B	SE	Wald	df	*p*-value	HR	95% CI for HR	
							**Lower**	**Upper**
Therapy group	–0.183	0.470	0.151	1	0.698	0.833	0.332	2.094
Number of brain metastases	0.731	0.348	4.414	1	0.036	2.077	1.050	4.109
Fractionation	1.593	0.708	5.065	1	0.024	4.917	1.228	19.682
Co-existence of systemic disease	0.096	0.451	0.045	1	0.832	1.100	0.455	2.663

B: regression coefficient (beta); SE: standard error; df: degrees of freedom; HR: hazard ratio; CI: confidence interval.

Of the 99 patients included in the analysis, 16 (16.2%) patients presented with acute grade 3+ toxicity. Out of the 16 patients, seven (11.1%) did not receive systemic treatments concurrently, and nine patients (25%) were in the concurrent systemic group. Table 4 shows acute toxicity grade ≥ 3 by systemic therapy group. A *χ*^2^ test for association was conducted between toxicity and therapy group for all patients included in the analysis. All expected cell frequencies were greater than five. There was no statistically significant association between toxicity and therapy group (*p* = 0.071). As for late toxicity, only one case of symptomatic radionecrosis was reported. This occurred in a patient after SRS delivered subsequently to the combination of WBRT with ICI because of tumor progression. Radionecrosis was diagnosed a year after SRS by tissue biopsy.

**Table 4:** Grade ≥ 3 acute toxicity by systemic therapy group.

Therapy group		GR ≥ 3 toxicity		Total	*p*-value
		**No**	**Yes**		
Non systemic	Number of patients	56	7	63	0.071
	% within therapy group	88.9%	11.1%	100.0%	
Systemic	Number of patients	27	9	36	
	% within therapy group	75.0%	25.0%	100.0%	
Total	Number of patients	83	16	99	
	% within therapy group	83.8%	16.2%	100.0%	

GR: grade.

## Discussion

A primary goal of our study was to monitor toxicity of the combined administration of WBRT with systemic agents. Only two patients included in the study did not complete WBRT, neither of whom received antineoplastic treatments concomitantly. This confirms that urgent hospitalization during WBRT is rare. Overall, serious acute toxicities were infrequent, and no statistically significant differences were found between patients treated with WBRT only and patients receiving systemic treatment concurrently. Serious adverse events in our study were reported in 16.2% of patients, which is consistent with the 19.4% and 20.3% treatment-related toxicity ≥ grade 3 reported in the phase III clinical trial of hippocampal-avoidance WBRT in the investigational and in the comparator arm, respectively [18, 19]. In another phase III clinical trial comparing the addition of thalidomide to WBRT, the rate of serious adverse effects in the standard arm was 11.96%, which is similar to the rate of 11.1% in our study for the group of patients not receiving systemic treatments concurrently [20]. Still, a tendency for an increased incidence of serious adverse events in patients receiving systemic treatments reported in our study warrants further investigation.

OS from BM improves with time. In our study, median OS was 6 months, which correlates well with the contemporary literature [18, 21]. In a retrospective cohort of 148 patients treated for BM, the median OS for those treated with WBRT was 6.9 months, which is similar to our results [22]. In the overall study population, concomitant systemic treatments and presence of systemic disease were found to be independent prognostic factors of OS. The presence of extracranial metastases is a known negative prognostic factor of OS for BM patients. Age and PS, other common prognostic factors, did not reach statistical significance in our study [21]. We also found no correlation between neurological symptoms and patients’ prognosis. Recent registry studies have found neurological symptom burden to negatively impact prognosis of BM patients from different primaries, namely melanoma, NSCLC, SCLC and breast cancer [23–26]. As prognostic factors vary by primary diagnosis [27], and systemic treatment [28], a possible explanation of our difference in results is that our analysis was not tumor- and treatment-specific.

Similar to our results, other studies have also found a positive association when combining systemic treatments with WBRT in terms of OS [29, 30]. Even a study with short-term survivors from BM, who presumably get less benefit from treatments, showed that the addition of systemic treatments was favorable to patients’ survival [31]. However, some studies suggest a potential deleterious effect of the concurrent use of certain systemic treatments with WBRT [32, 33]. Yet, these two studies included temozolamide as a systemic treatment, not frequently used today concurrently with WBRT, and which was not received by any patient in our study. A meta-analysis of RCTs on the impact of chemotherapy given concurrently with WBRT to BM patients from NSCLC showed an increase in intracranial response rate, with no OS benefit, and a detrimental effect on toxicity [34]. In our study, however, almost half of the patients treated with concurrent systemic treatments received chemotherapy.

A significant finding of our study is the improvement in OS with the concurrent use of systemic treatments. Most patients in our study were treated with systemic treatments, including ICI, anti-HER2 drugs, hormone treatment, targeted agents, and/or chemotherapy, either within two weeks of WBRT, or with a longer interval between treatments. Only a third received WBRT concurrently, i.e., within two weeks, with antineoplastic drugs. Therefore, the benefit shown in our study is a result of this proximity of treatments. Immunotherapy has been mainly studied in combination with SRS [35, 36], and only a few small studies have investigated the combination of immunotherapy and WBRT [37, 38]. Yet, as melanoma patients are referred to other radiotherapy units in Athens, they were absent from our study. All other main primaries were represented in our study. Similarly, very few studies have examined WBRT with anti-HER2 drugs, even though many studies have shown their combination with SRS to be, generally, safe [39, 40]. A small retrospective study also concluded that both chemotherapy and anti-HER2 treatment after WBRT improved OS of HER2 positive breast cancer patients [41]. Concerning targeted agents, only two NSCLC patients were treated concurrently with TKIs and WBRT in this study. Rash, a common side effect of TKIs, combined with radiation dermatitis, a common side effect of WBRT, could have refrained medical oncologists from giving TKIs within two weeks of WBRT [42]. Our study cannot draw safe conclusions for this subgroup of patients.

Fractionation was found to be statistically significantly correlated to TTICP, with the more prolonged treatment increasing time without intracranial relapse. Previous retrospective studies on NSCLC patients comparing the five- and ten-fraction regimen had failed to show an improvement of OS with prolonged fractionation, and, thus, these two treatments are considered equivalent, and their impact on OS was not examined in our study [43]. Still, previously, there have been studies showing a difference even in OS with unconventional fractionations [44]. The impact of fractionation on TTICP found in the present study merits further investigation, as prevention of neurological death is a key goal of intracranial treatments. In the modern era of more potent systemic treatments, achieving better control of systemic disease, it is important to re-examine the impact of different fractionations on intracranial control rates and intracranial control duration. The difference in neurocognitive deterioration with different fractionations is also worth examining but was beyond the scope of our study.

The number of BM was found to be associated with an increase in the time of intracranial control, which seems to be counterintuitive. This could be explained by the fact that at the time of the study, most patients were diagnosed with BM when symptomatic. Therefore, patients with a small number of metastases could more commonly present with larger lesions than patients with numerous metastases. Other studies have found that the cumulative metastatic intracranial volume is a more robust prognostic factor of intracranial response to local treatments [45]. The use of the number of metastases in our study, instead of the cumulative volume, could limit the generalizability of our results. Also, this finding could result from the small number of patients with available imaging follow-up, which is the primary limitation of our study concerning TTICP. Yet, the limited number of available follow-up MRI scans is a common problem encountered in studies of WBRT, intrinsic to the nature of the disease [46].

Major limitations of our study include the relatively small number of patients and the pooled analysis of patients with different primary diseases and who are treated with various systemic agents. This did not allow us to further analyze subgroups of our patient population by primary or by type of systemic therapy. Moreover, all observational studies are subject to information bias, such as recall bias, and loss to follow-up bias. Typical of observational studies, selection bias may also be present in our study [47]. The attribution of systemic therapies concurrently or not to WBRT was at the discretion of the medical oncologist. The decision was based on a variety of factors, including comorbidities, burden of systemic disease, previous treatments, and fear of toxicity for newer systemic treatments. These limitations should be considered when interpreting the results of our study.

Data concerning the combination of contemporary systemic treatments with WBRT is scarce, and often contradictory. The combination of new-generation systemic agents, either immunotherapy or targeted agents, with radiotherapy has been studied preferentially with SRS, available in most academic centers. Many patients with BM are treated with WBRT because of a larger volume of intracranial disease, because of the unavailability of stereotactic treatments locally, or because of their poor prognosis. In the future, the role of WBRT could be reestablished as the main local treatment for BM patients, as new planning techniques that spare the hippocampus, the corpus callosum, the fornix, and the amygdala, which are the substructures of the brain associated with neurocognition, are actively being investigated [48]. It is, therefore, important to continue studying WBRT as it is used nowadays, with the use of modern systemic treatments. Our study reports on the results of a real-world setting where WBRT was delivered with and without the concomitant use of systemic treatment, and showed safety and efficacy, even for patients who were long-term survivors. Our results suggest a possible detrimental effect when delaying the initiation or when discontinuing systemic treatments for WBRT. Prospective trials that are drug-specific are necessary to confirm safety and trials that are also primary-specific are needed to investigate the efficacy of the combined treatment.

## Abbreviations

BM: brain metastases
CI: confidence interval
ICI: immune checkpoint inhibitor
OS: overall survival
RCTs: randomized controlled trials
SRS: stereotactic radiotherapy
TKI: tyrosine kinase inhibitor
TTICP: time to intracranial progression
WBRT: whole-brain radiotherapy
*χ* ^2^: chi-square

## Declarations

### Availability of data and materials

The datasets supporting the findings of this study are available from the corresponding author upon reasonable request.

### Copyright

© The Author(s) 2026.
